# Experimental and Simulation Study for the Influence of Thermal Pre-Deformation on Subsequent Aging Precipitation Kinetics of Al-Zn-Mg-Cu Alloy

**DOI:** 10.3390/ma15134634

**Published:** 2022-07-01

**Authors:** Qian Sun, Sha Yu, Hong Wang, Huijuan Ma, Huanhuan Li, Zhili Hu

**Affiliations:** 1 School of Automotive Engineering, Wuhan University of Technology, Wuhan 430070, China; sunqian20180118@163.com (Q.S.); yusha212729@163.com (S.Y.); wanghong94011@163.com (H.W.); 2 Hubei Collaborative Innovation Center for Automotive Components Technology, Wuhan University of Technology, Wuhan 430070, China; 3 Hubei Research Center for New Energy and Intelligent Connected Vehicle, Wuhan University of Technology, Wuhan 430070, China; 4 School of Mechanical and Electrical Engineering, Wuhan Institute of Technology, Wuhan 430070, China; 20060302@wit.edu.cn; 5 Hubei Provincial Key Laboratory of Chemical Equipment Intensification and Intrinsic Safety, Wuhan 430205, China

**Keywords:** thermal deformation, aging precipitation kinetics, modeling, dislocations, Al-Zn-Mg-Cu alloy

## Abstract

Deformation and heat treatment are important means to strengthen aluminum alloys. However, the influence mechanism of pre-strain on aging precipitation kinetics and its effect on mechanical properties are still not clear. In this work, uniaxial isothermal tensile tests with different strains and artificial aging treatments for Al-Zn-Mg-Cu alloys have been carried out. Then, a model describing the precipitates kinetic behavior has been developed to investigate the effect of thermal pre-strain on subsequent aging precipitation kinetics and peak aging microhardness based on the microstructure characterization by TEM, SAXS and XRD tests. In addition, the role of dislocations on the aging precipitation kinetics is also explored. The experimental results show that the peak aging microhardness of the Al-Zn-Mg-Cu alloy reveals a tendency to decrease and increase and then the peak aging time firstly decreases and then keeps almost constant with the increase in the strain. The calculations demonstrate that the precipitate average size almost remains unchanged, while the precipitate volume fraction decreases and then increases with the increase in strain, which is consistent with the change in peak aging microhardness. It also indicates that dislocations can promote precipitate nucleation and growth, while the actual effect depends on the dislocation density, which is closely dependent on the pre-deformation condition, especially for the precipitate nucleation. In particular, when the dislocation density after thermal pre-deformation is not enough, it will slightly inhibit precipitate nucleation but promote precipitate growth, which could shorten the peak aging time, with the peak aging strength being guaranteed.

## 1. Introduction

To alleviate the problem of global warming, fuel economy and emissions control is necessary. One solution to the above issues is to reduce the weight of aerospace and auto-mobile artifacts by utilizing lightweight metals, such as aluminum alloys. The application of the hot-stamping technique has emerged to overcome the low form ability and guarantee the dimensional accuracy of aluminum alloys [1,2,3]. The hot stamping process of aluminum alloys can be characterized by relatively high deformation temperatures and high strain rates, which will definitely affect the subsequent aging precipitation and the final mechanical properties. Therefore, it is necessary to study the effect of thermal deformation on the following artificial aging and its effect on the final mechanical performance of aluminum alloys for evaluating the applicability and optimizing the process parameters of hot stamping.

The effect of pre-deformation on subsequent artificial aging has been extensively studied for many heat treatable aluminum alloys [4,5,6,7,8,9,10,11]. The results show that pre-deformation can be positive or negative on the mechanical properties of aluminum alloys depending on the alloy composition, precipitate types and deformation parameters [9,12,13,14,15]. For the Al-Zn-Mg-(Cu) system or alloys based on Al-Zn-Mg-(Cu), which play an important role for light-weight structural applications, in particular the aircraft industry, the effect of pre-deformation on the precipitation process and mechanical properties after aging has also been studied [9,12,15,16]. Some researchers have put forward that pre-deformation will not promote the mechanical properties when aging at higher temperatures (120–160 °C) for 7XXX alloys. For example, for the AA7108 and AA7030 alloy, the samples with 10% pre-deformation exhibited lower yield strength than that of the T6 samples [15], while the strength of the alloy maintained the same level as the T6 treated sample in 7A04 alloys when larger pre-deformation (50%) was applied [16].

Considerable research has been carried out to explore the influence mechanism of pre-deformation on subsequent artificial aging, and a widely accepted view is that pre-deformation obviously influences the subsequent aging precipitation kinetics [17,18,19,20,21,22]. For example, many experimental results have demonstrated that pre-deformation can promote the coarsening of precipitates phases [12,17,18] and also modeling of the precipitation with nanoscale to micron scale during the aging of an alloy has attracted considerable attention in past decades [23,24,25,26] in order to clarify the above issues. It has been discussed that the growth of precipitates on dislocations is faster than in the bulk and the presence of dislocations generally results in an acceleration of the global precipitation kinetics, since the limiting mechanism for precipitate growth is bulk diffusion towards dislocations rather than pipe diffusion along dislocations [19] or is to induce short-circuit diffusion paths for solutes and then modify bulk precipitation kinetics [20].

However, the role of dislocations generated by deformation on the precipitation kinetic of pre-deformed alloys is still controversial. On the one hand, some researchers reported that dislocations have no catalyzing effect on the nucleation of precipitates [21] and even inhibit the nucleation of precipitates [22], while others proposed that dislocations promoted the nucleation of precipitates [11,20,27,28]. On the other hand, some studies have been conducted to investigate the age hardening characteristics of the pre-deformed Al-Zn-Mg-Cu alloy [23,29,30,31,32]. However, most of the studies have focused on the effect of cold deformation on the subsequent aging precipitations. Studies about the influence of thermal deformation on the following aging precipitations of the Al-Zn-Mg-Cu alloy, which is usually applied to hot forming such as hot stamping process, are few in number [33].

Thus, in order to simulate the hot stamping process and investigate the effect of thermal pre-deformation on the subsequent aging precipitation of the Al-Zn-Mg-Cu alloy, uniaxial isothermal tensile tests were carried out with the complete solution treated aluminum alloy and then artificial aging treatments at 120 °C were conducted in this work. In addition, a model calculating the aging precipitation that takes the concurrent actions of the nucleation, growth, coarsening of the precipitates into account has been utilized to analyze the influence mechanism of thermal pre-deformation on the aging precipitation kinetics and explore the role of dislocations on aging precipitation kinetics of the Al-Zn-Mg-Cu alloy.

## 2. Materials and Methods

AA7075-T6 aluminum alloy sheets provided by Southwest Aluminum Company, which is a kind of Al-Zn-Mg-Cu alloy with 1.5 mm thickness, were used as the raw material, whose chemical compositions are listed in Table 1. Solution treatment and thermal tensile deformation tests with different strains have been carried out by the Gleeble-3500, following by water quenching and resulted in a fibrous structure with an average grain size of approximately 16~18 μm [34].

**Figure 1:**
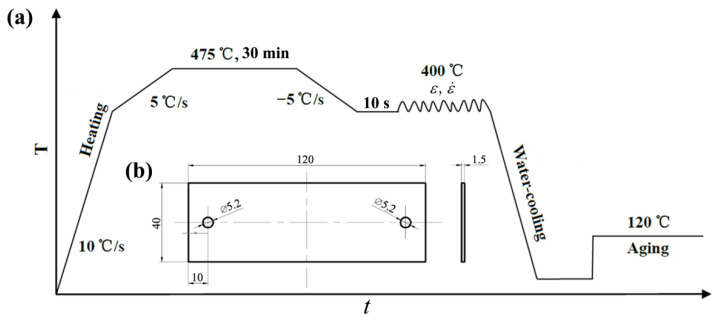
(**a**) Schematic diagram of thermal simulation experiment and subsequent aging process; (**b**) specimen size for thermal tensile test.

The detailed process design is shown in Figure 1a. The samples were first heated to 400 °C with a heating rate of 10 °C/s. Then, the samples were heated to 475 °C with a heating rate of 5 °C/s, and held for 30 min to ensure a complete solid solution in accordance with standard YS/T 591-2017. After that, the temperature dropped to 400 °C at a cooling rate of −5 °C/s. The reason for heating and cooling with the second stage of ±5 °C/s is to avoid over-heating or over-cooling and stabilize the temperature as soon as possible.

At 400 °C, the tensile deformation was performed with a different strain (0~0.25). The size of the samples for thermal simulation experimental was 120 mm × 40 mm × 1.5 mm and its length direction was along the rolling direction of the blank, as observed in Figure 1b. After that, the deformed samples were water quenched and then specimens with size of 10 mm × 10 mm × 1.5 mm were cut from the middle of the deformed samples to carry out 120 °C artificial aging with different aging times (2~28 h). The detailed deformation and aging parameters can be observed in the Table 2.

After aging, Vickers hardness (HV) tests at five different positions of specimens were carried out to measure the average hardness change with different aging times. Meanwhile, the dislocation density of the specimens was tested by XRD. After the hardness test, specimens were ground and polished for microstructure characterization. Then, the distribution of precipitates was analyzed by TEM and SAXS.

X-ray diffraction (XRD) measurements were performed with a Cu Kα radiation at voltage of 40 kV and current of 200 mA (Rigaku D/max 2500PC). The scanning ranged from 35° to 80° with a scanning speed of 1.5°/min. Each alloy was measured 3 times in a different position to obtain an average value.

The small angle X-ray scattering (SAXS) experiments were carried out on the Xeuss 3.0. All SAXS samples were thinned to ~80 μm. The scattering phenomenon that occurred within a small angle range of 2° to 5° close to the original beam by irradiating the sample with X-rays was used to analyze the average radius and volume fraction of the precipitates. A Cu Kα source was used, giving a wavelength of 1.54 Å (8 keV) with a beam size of 200 μm × 200 μm. Acquisition was carried out with a 2D CCD camera, which provided a range of scattering vectors Q (0.03, 49) nm^−1^, giving access to all types of precipitates present in the microstructure, from GP zones of about 1 nm diameter to coarse precipitates of about 200 nm diameter. The measured SAXS data were corrected for the incident beam intensity, transmission factor, the thickness of the sample, background noise, and then converted to absolute units by measuring a glassy carbon standard sample.

After the raw data were deducted and normalized, we obtained an SAXS curve generated by the difference in electron density between the precipitate and the matrix. The Guinier radius ($R_{g}$) of the precipitates can be calculated as follows from the IQ^2^–Q curve [35]:

(1) $$R_{g}=\frac{\sqrt{3}}{Q_{\max}}$$

where $Q_{\max}$ is the position of the maximum of IQ^2^ vs. Q plot. Then, the volume fraction of the precipitates can be calculated based on Equation (2) using the integrated intensity $Q_{0}$ [35], which is as follows:

(2) $$Q_{0}=\int_{0}^{\infty }I(Q)Q^{2}dQ=2\pi^{2}{\left(\Delta \rho \right)}^{2}f_{v}(1-f_{v})$$

where $f_{v}$ is the volume fraction of the precipitates, I(Q) is the scattering intensity and Δρ is the electron density contrast between the precipitates and matrix.

Thus, the number density $N_{v}$ of precipitates can be estimated based on Equation (3), which is as follows:

(3) $$N_{v}=\frac{\mu f_{v}}{2\pi R_{g}^{3}}$$

where μ is the ratio between the diameter and the thickness of the disc-shaped precipitates. More details about the SAXS can be found in reference [35].

Specimens for transmission electron nicroscope (TEM) characterization were prepared directly from the SAXS specimens by grinding them to a thickness of ~50 μm and then, 3 mm diameter thin samples were punched out from thin foils for ion thinning, using 30% HNO_3_ in methanol at −30 °C, with an operating voltage of 20 V. TEM samples were examined in a TECNAI G2 F30 transmission electron microscope.

## 3. Results

### 3.1. Influence of Thermal Pre-Deformation on Microhardness of Al-Zn-Mg-Cu Alloy during Subsequent Aging

**Figure 2:**
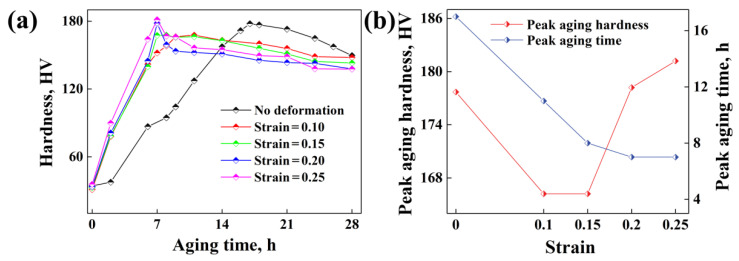
(**a**) Vickers hardness of samples with aging time under different strains; (**b**) peak aging hardness and peak aging time for samples with different strains.

The Vickers hardness of the sample changes with aging time with different strains (deformation temperature 400 °C and strain rate 0.1 s^−1^) is shown in Figure 2a.

As the strain changes from 0 to 0.10, 0.15, 0.20 and 0.25, the corresponding maximum hardness changes from 177.7 HV to 166.2 HV, 166.2 HV, 178.2 HV, 181.2 HV, which appears at different aging times with 17 h, 11 h, 8 h, 7 h and 7 h, respectively, as shown in Figure 2b. It shows that the peak-aging hardness decreases and increases with the increase in strain, while the peak-aging time decreases and then almost remains constant with the increase in strain. Considering that the microhardness and peak aging time of the specimen with strain 0.25 is approximately consistent with that of the specimen with strain 0.20, only specimens with strain 0~0.20 have been prepared for XRD, SAXS and TEM tests.

### 3.2. Effect of Thermal Pre-Deformation on Aging Precipitates Distribution of Al-Zn-Mg-Cu Alloy

**Figure 3:**
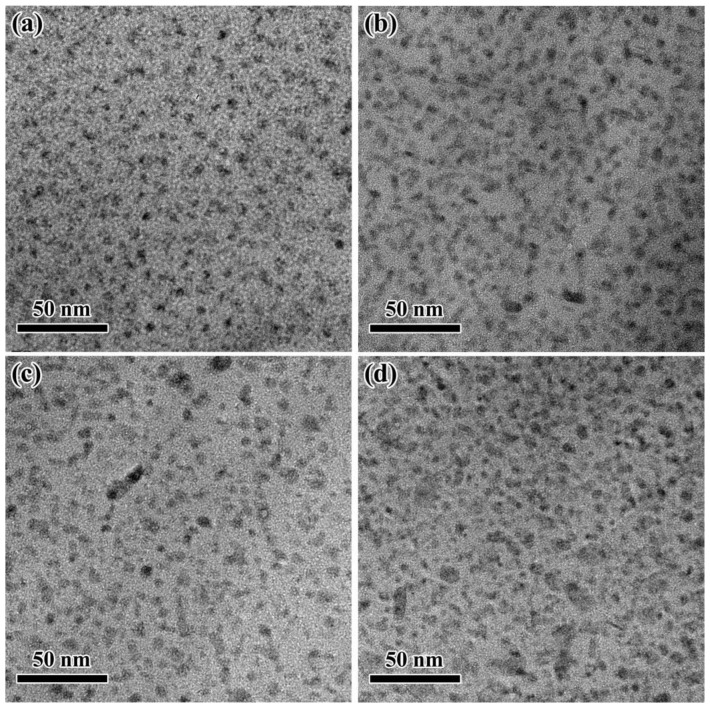
Lower magnification TEM images of precipitates after aging at 120 °C for 28 h with different strains: (**a**) 0; (**b**) 0.10; (**c**) 0.15; (**d**) 0.20.

**Figure 4:**
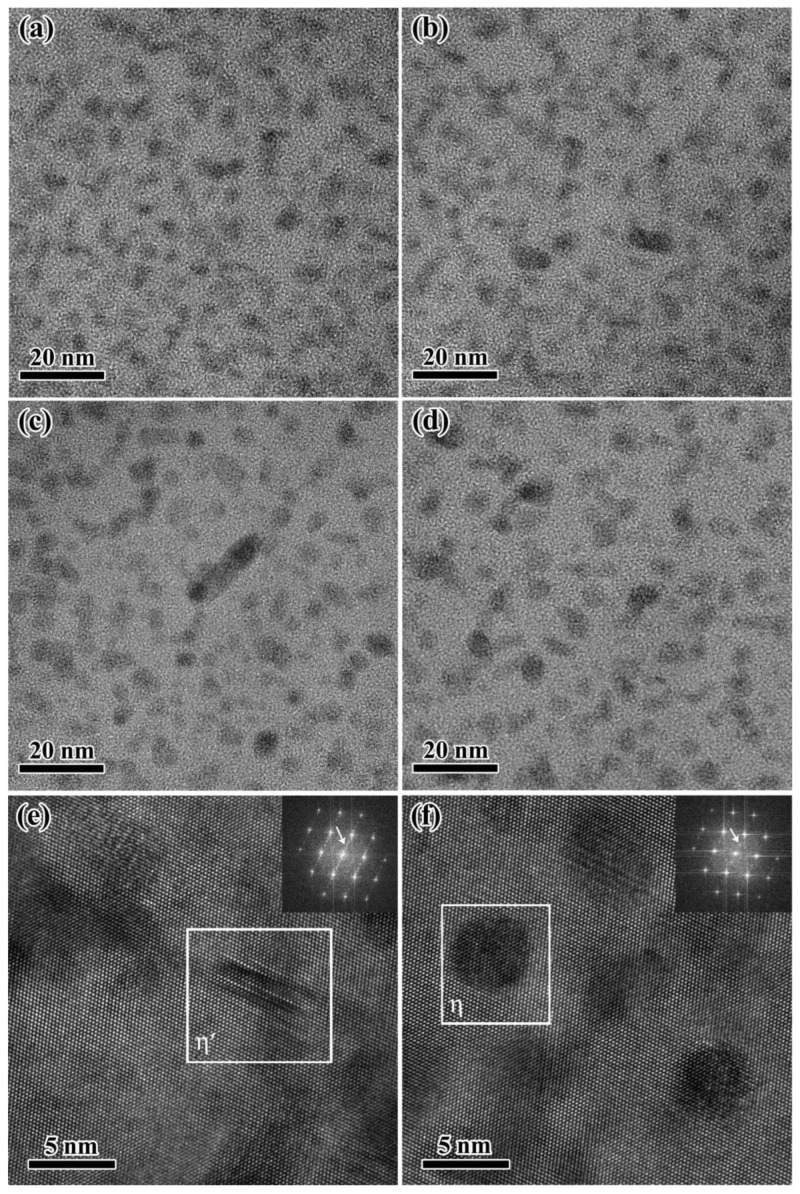
Higher magnification TEM images of precipitates after aging at 120 °C for 28 h with different strains: (**a**) 0; (**b**) 0.10; (**c**) 0.15; (**d**) 0.20; <011>_Al_ FFT of the (**e**) rod-like η′ phase and (**f**) disk η phase.

The microstructure determines the final mechanical properties of the aging Al-Zn-Mg-Cu alloy, which mainly including grains, sub-grains and precipitated phases. In our previous work [34], it has been demonstrated that no re-crystallization occurred during thermal deformation and the average grain size after 28 h aging at 120 °C basically remains constant with different strains. Thus, here we focus on the aging precipitates of the Al-Zn-Mg-Cu alloy. Figure 3 shows the TEM images of precipitates for specimens after isothermal heat treatment at 120 °C for 28 h with different strains. The distribution of the precipitates seems to show a tendency of gradually coarsening with the strain, as shown in Figure 3. The morphology of the precipitates with higher magnification is shown in Figure 4a–d. According to Figure 4e,f, it indicates that there mainly exists η′ phase and η phase [36] for specimens of the Al-Zn-Mg-Cu alloy at 120 °C for 28 h with different strains.

**Figure 5:**
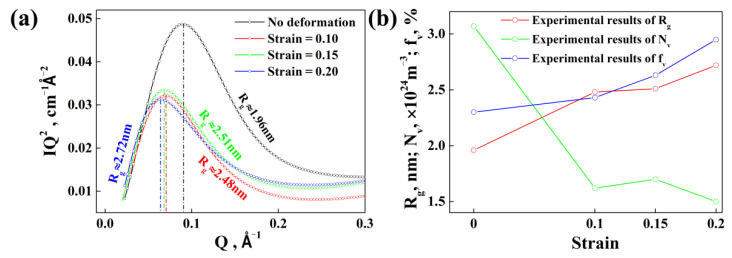
(**a**) Guinier radius $R_{g}$ of specimens with different strains; (**b**) experimental results for average radius $R_{g}$, volume fraction $f_{v}$, and number density $N_{v}$ of precipitates for specimens after isothermal heat treatment at 120 °C for 28 h with different strains.

The quantitative information of nanoscale precipitates is evaluated by the SAXS method [35] for specimens with different strains. The results are shown in Figure 4. It can be found that the $R_{g}$ of specimens with different strains is 1.96 nm, 2.48 nm, 2.51 nm and 2.72 nm, respectively, as shown in Figure 5a. Then, the volume fraction $f_{v}$, and number density $N_{v}$ of precipitations can be calculated. The calculation results show that the volume fraction $f_{v}$ increases while the number density decrease with the strain, as shown in Figure 5b.

## 4. Model

The aging precipitation evolution for the Al-Zn-Mg-Cu alloy is a result of the concurrent actions of the nucleation, growth, coarsening, and structural transformation of the precipitates with the following usual precipitation sequence [29]: solid solution → GP zones → metastable η′ → stable η (MgZn_2_). The size and distribution of the precipitates mainly depend on the aging process. To understand the detailed evolution process of the precipitates for the Al-Zn-Mg-Cu alloy during aging, a model is developed to calculate the kinetic behavior of the precipitates. In order to calculate the precipitation kinetics of an aged aluminum alloy during continuous aging and keep the model at a reasonable level of simplicity and number of parameters, several simplifications need to be emphasized, which are as follows: (1) the aluminum alloy used for experiments is simplified as Al-Zn-Mg-Cu ternary alloy and the effect of other elements on the precipitation kinetics during cooling and aging should be considered through the value of the activation energy for the precipitates nucleation and the diffusivity of the solute; (2) considering that the atomic concentration ratio of solute Zn/Mg for the raw materials is 1/1.3 while the atomic concentration ratio of solute Zn/Mg for MgZn_2_ is 2, and the diffusion coefficients of solute Mg and Zn are of basically the same order of magnitude [37,38], the equivalent diffusion coefficient is dependent on that of solute Zn; (3) the GP zones are not considered directly in this model, but enter the model as nucleation sites for the precipitate and are taken into account through the value of the activation energy for precipitate nucleation [23,26].

To predict the precipitation kinetics of an aging aluminum alloy by using the population dynamics model [39], which is usually applied for a growing or shrinking spherical particle, we assume the precipitate is a pseudo sphere with the radial growth rate being proportional to the thick-directional growth rate for the disc-shaped or flake precipitates of the Al-Zn-Mg-Cu alloy [40]. Then, a function f(r,t) is defined to describe the radius distribution of the precipitated phases. f(r,t)dr represents the number of the precipitates per unit volume in a radius range between r and r+dr at time t. According to this definition, the number density $N_{v}$, average radius $R_{g}$ and volume fraction $f_{v}$ of the precipitates are given by the following equations [39]:

(4) $$N_{v}=\int_{0}^{\infty }f(r,t)dr$$

(5) $$R_{g}=\left(1/N_{v}\right)\int_{0}^{\infty }rf\left(r,t\right)dr$$

(6) $$f_{v}=\left(2\pi /\mu \right)\int_{0}^{\infty }r^{3}f\left(r,t\right)dr$$

It might be reasonable to assume that the precipitates show a homogeneous distribution in the matrix alloy during the pre-deformation and subsequent aging process. Under such conditions, f(r,t) satisfies the following equation [39]:

(7) $$\frac{\partial f\left(r,t\right)}{\partial t}+\frac{\partial }{\partial r}\left(vf\left(r,t\right)\right)={\frac{\partial I}{\partial r}|}_{r=r^{*}}$$

where the first term describes the time dependence of the precipitate radius distribution function; the second term describes the contribution of the growth/shrinkage of the precipitates to the variation in the distribution function; the term on the right-hand side is the source term stemming due to nucleation of the precipitates. I is the classical homogeneous nucleation rate of the precipitates and v is the diffusional growth/dissolution rate of the precipitates.

The classical homogeneous nucleation rate of the precipitates (I) can be calculated by Equation (8) [23,26], which is as follows:

(8) $$I=Z\beta^{*}N_{0}\exp\left(\frac{-\Delta G_{c}}{k_{B}T}\right)\cdot \exp\left(\frac{-\tau }{t}\right)$$

with the abbreviation

$$\beta^{*}=\frac{4\pi {\left(r^{*}\right)}^{2}DC_{m}}{a^{4}}$$

$$\Delta G_{c}=\frac{16}{3}\pi \frac{\sigma^{3}}{{\left(\Delta G_{v}\right)}^{2}}$$

$$\tau =\frac{1}{2\beta^{*}Z}$$

where Z is the Zeldovich nonequilibrium factor (≈0.05 [20]); $\beta^{*}$ is the attachment rate of atoms to the nucleus of the critical radius; $N_{0}$ is the number density of atoms by unit volume for the precipitated phase; τ is the incubation time; a is the average lattice parameter of the nucleus and the matrix; $\Delta G_{c}$ is the activation energy for the nucleation of the precipitates; $\Delta G_{v}=-\frac{k_{B}T}{v_{\alpha }}\ln\left(\frac{c_{m}}{c_{eq}}\right)$ is the gain in free energy per volume on nucleation [23]; $c_{m}$ is taken as the concentration product of the atomic concentrations of Mg and Zn in the matrix, $c_{eq}$ is taken as the concentration product of the equilibrium atomic concentrations of Mg and Zn; $k_{B}$ is the Boltzmann’s constant; T is the absolute temperature; $v_{\alpha }$ is the average volume per atom in the nucleus; $D=D_{0}\cdot (-Q_{d}/(RT))$ is the diffusional coefficient of the solute Zn for the precipitation of the precipitated phases [37]; $r^{*}=-\frac{2\sigma }{\Delta G_{v}}$ is the critical nucleation radius for the precipitates; σ is the interfacial energy between the precipitate and the matrix.

The growth rate (v) of the precipitates can be calculated by Equation (9) [23,26,39], which is as follows:

(9) $$v=\frac{D}{r}\cdot \frac{C_{m}-C_{I}}{C_{P}-C_{I}}+\frac{I}{N}(\alpha \cdot r^{*}-r)$$

where $C_{m}$ is the mean molar concentration of solute Zn in the matrix; $C_{eq}$ is taken as the equilibrium molar concentration of solute Zn in the matrix; $C_{p}$ is the molar concentration of solute Zn in precipitates; $C_{I}=C_{eq}\exp(\frac{2\sigma v_{\alpha }}{rk_{B}T})$ is the molar concentration of solute Zn at the precipitate/matrix boundary; α is a constant. The numerical solution method for above model can be found in reference [39].

The driving force for the nucleation of the precipitates, as well as the solubility of the MgZn_2_ phase in the Al-rich phase for Al-Zn-Mg-Cu alloys, can be calculated based on Equation (10) [40,41].

(10) $${\left(c_{Zn}\right)}^{2}\cdot c_{Mg}=A_{0}\cdot \exp\left(\frac{-\Delta H_{sol}}{RT}\right)$$

where $c_{Zn}$ and $c_{Mg}$ are equilibrium atomic concentrations of alloying elements Zn and Mg in the Al-rich matrix phase; $A_{0}$ is a constant; $\Delta H_{sol}$ is the dissolution enthalpy of the precipitates. According to the sum of the equilibrium atomic concentration of Mg and Zn, which equals to 1.0% at 160 °C and 5.4% at 400 °C [26], as well as the experimental results for the specimen without pre-deformation, the dissolution enthalpy of the precipitates ($\Delta H_{sol}$) can be fixed at about 63.8 kJ/mol based on Equation (10). Other main thermo-physical parameters used in the calculation are shown in Table 3.

## 5. Calculation and Discussion

### 5.1. Influence of Thermal Pre-Deformation on the Subsequent Aging Precipitation Kinetics of Al-Zn-Mg-Cu Alloy

It has been widely accepted that pre-deformation can promote the subsequent aging kinetics of precipitates for aluminum alloys. In detail, according to Equations (8) and (9), pre-deformation might change the interfacial energy between the precipitates and aluminum matrix (σ) [43]. In addition, it can also promote the diffusion of solute atoms [17,21,44] ($D=D_{0}\cdot (-Q_{d}/(RT))$), and then influence both the growth and the nucleation of precipitates. Thus, for specimens pre-deformed with different strains, three typical parameters of precipitates, such as the average radius, number density and volume fraction of precipitates for samples, have been calculated, utilizing the established model with two key parameters (σ and D) being fitted simultaneously according to the corresponding experimental results shown in Figure 5.

**Figure 6:**
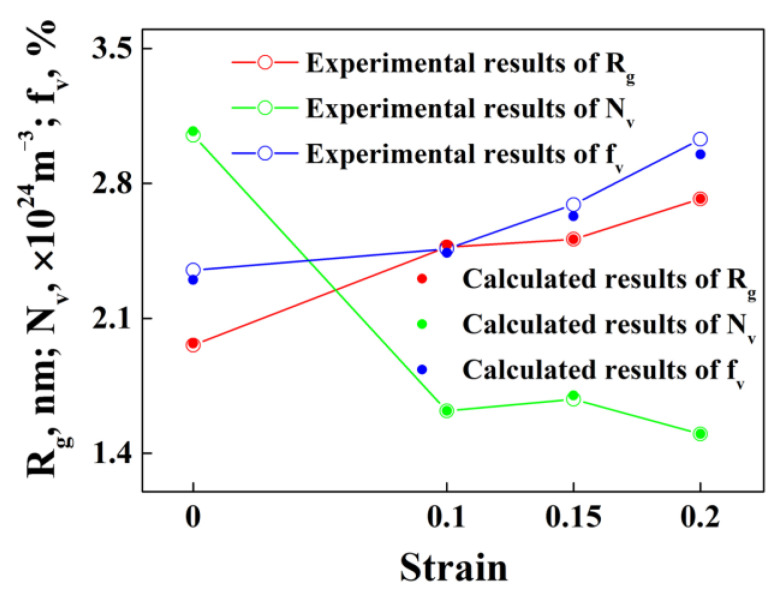
Calculated and experimental values for the average radius, number density and volume fraction of the precipitates for alloy samples with different strains.

Figure 6 shows the calculated and experimental values for the average radius, number density and volume fraction of the precipitates for specimens with different strains. It can be observed that the calculated results are basically consistent with the experimental results, which demonstrates that the calculated results are reliable.

**Figure 7:**
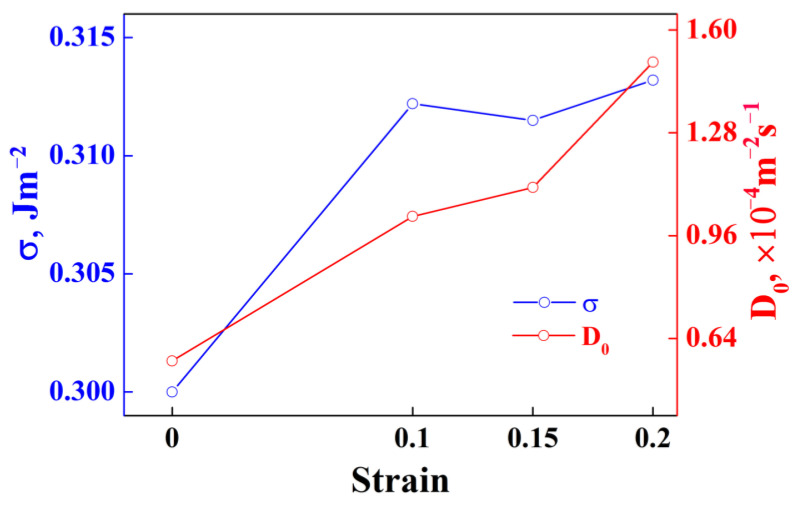
Values of σ and $D_{0}$ for the alloy samples with different strains.

In addition, the fitting results for σ and $D_{0}$ are shown in Figure 7. It shows that both σ and $D_{0}$ show a tendency to increase with the increase in strain. Combined with Figure 6 and Figure 7, it can be observed that thermal pre-deformation slightly inhibits the nucleation of precipitates, while promotes the growth of precipitates during subsequent aging.

**Figure 8:**
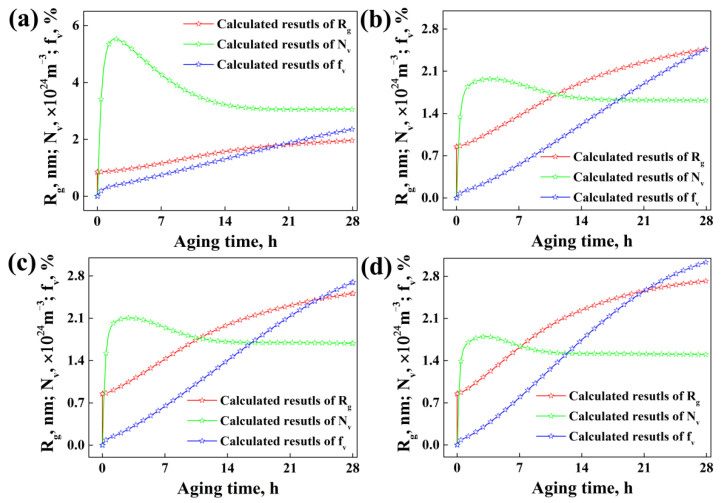
Average radius, number density and volume fraction of precipitates during aging for the alloy samples with different strains: (**a**) 0; (**b**) 0.10; (**c**) 0.15; (**d**) 0.20.

At the same time, according to the calculated results shown in Figure 8, the evolution process of the average radius, number density and volume fraction of precipitates for specimens with different strains are similar. It is worth noting that the average radius and volume fraction of precipitates for all the specimens increase with the increase in aging time during the whole aging process, while for the number density of precipitates, it increases dramatically (<2 h) first, then decreases gradually (2~10 h) at the early stage of aging, and finally remains almost constant (10~28 h), along with the increase in aging time. This might be related to the theory of Ostwald ripening or LSW coarsening [20,21,45], another form of precipitation growth in addition to diffusion growth. Ostwald ripening usually occurs when the solute supersaturation of the matrix is relatively low, which will not meet the solute supply for the continued growth of all the nucleus and then a large number of precipitates with smaller radius, such as GP zones or η′, cannot grow continually and they would be consumed by other precipitates with greater radius, such as η′ or η. Hence, although the average radius or volume fraction of precipitates increases, the total number density of precipitates will decrease. It demonstrates that the precipitates probably grow in the way of Ostwald ripening in the early aging stage, and then grow in the way of pure diffusion in the later aging stage under the experimental conditions in this work.

**Figure 9:**
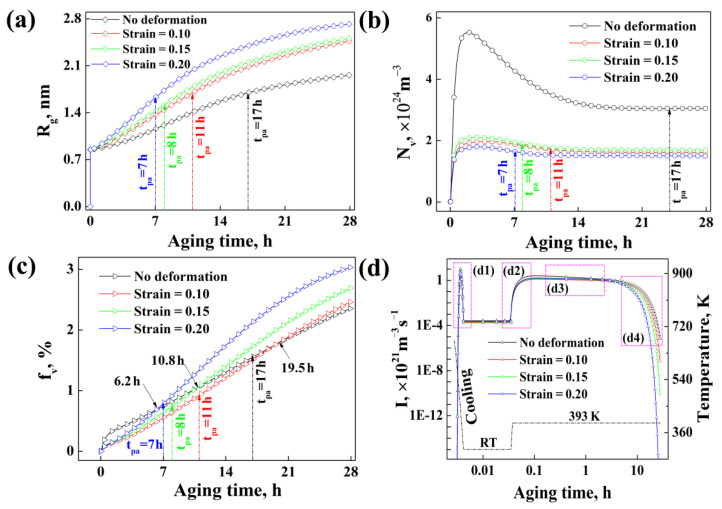
Average radius, number density, volume fraction and evolution of nucleation rate of precipitates changed with aging time for the alloy samples with different strains: (**a**) average radius; (**b**) number density; (**c**) volume fraction; (**d**) evolution of nucleation rate.

The precipitate average radius increases along with the increase in strain, as shown in Figure 9a; however, the precipitate number density basically decreases with pre-deformation, as shown in Figure 9b. Thus, the influence of strain on precipitate volume fraction is complicated during aging, since it is closely related to both the precipitate average radius and number density, as shown in Figure 9c. In the early aging stage, the precipitate volume fraction of specimens without deformations is higher than those of specimens with deformation, while in the later aging stage, the precipitate volume fractions of specimens with deformations exceed those of specimens without deformation. For example, the precipitate volume fraction of the specimens with strain 0.10, 0.15 and 0.20 exceeds that of the specimens without deformation at about 6.5 h, 10.8 h and 19.5 h during aging, respectively. Finally, the precipitate volume fraction of the specimens increases along with the strain when the aging time is 28 h.

**Figure 10:**
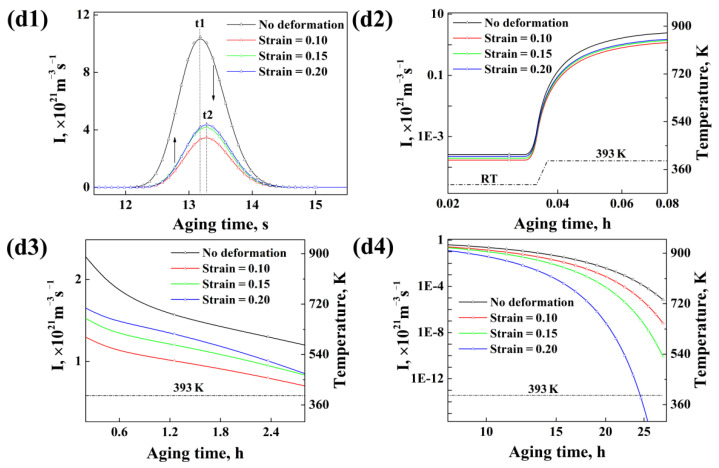
Details about the nucleation rate of precipitates for alloy samples with different strains (on the basis of Figure 9d).

In addition, compared with the change in precipitate number density for specimens with different strains, as shown in Figure 9b, we can observe that the Ostwald ripening phenomenon has been evidently weaken because of pre-deformation. This might be related to the following two factors: on the one hand, pre-deformation decreases the nucleation rate of precipitates, causing the decrease in the number density of precipitates at that instant during precipitation, as shown in Figure 9d and Figure 10, which will relieve the competition for solute consuming between precipitate nucleation and continual growth, thus weakening the extent of Ostwald ripening; on the other hand, pre-deformation promotes the diffusion of solute atoms, which will promote the growth of precipitation with long-distance diffusion and promote the diffusion-growth of precipitates. Hence, when the solute concentration in the matrix can meet the growth of all the nucleated precipitates after every nucleation process, Ostwald ripening will not occur and the number density of precipitates will not decrease during later aging. Therefore, it can be inferred that pre-deformation obviously affects the nucleation and the growth manner of precipitates, shortening the time needed for precipitate growth and changing the growth of precipitates from Ostwald ripening to pure diffusion growth.

Based on the above analysis, it can be deduced that the influence of pre-deformation on the average radius, number density and volume fraction of precipitates during the following aging is closely related with the nucleation behavior. The details for the influence of pre-deformation on the nucleation of precipitates discussed in the following section. Figure 10(d1–d4) from Figure 9d corresponds to different nucleation stages with water cooling as the beginning of the calculation (see Figure 1).

It can be found that, from the beginning of water cooling, the nucleation of precipitates begins (Figure 10(d1)). The maximum nucleation rate can reach (3~10) × 10^21^ m^−3^·s^−1^, but this nucleation process only lasts for a few seconds, since the temperature cools to room temperature (RT) quickly, which would restrict the growth of precipitates or it means that these nucleated precipitates are not stable. In addition, the average radius and volume fraction of the precipitates at this stage are also very small. After that, when the temperature is rising to 120 °C, the nucleation rate begins to increase gradually (Figure 10(d2)) and then remains approximately constant during heat preservation at 120 °C (Figure 10(d3)). The maximum nucleation rate for this stage can reach about (1~2) × 10^21^ m^−3^·s^−1^, which is just about one fifth of that during the cooling stage, while this nucleation process can last for about a few hours. Finally, the nucleation rate begins to clearly decrease when the aging time reaches about 10 h because the supersaturation of the matrix is not enough to support the nucleation of precipitates when the precipitates are growing, as shown in Figure 10(d4).

According to above discussion based on the experimental and calculated results, it can be inferred that the influence of thermal deformation on the subsequent aging precipitation kinetics is very complicated. Based on the experimental and calculated results in this paper, it can be concluded that, after thermal deformation, it will inhibit the actual nucleation behavior of precipitates and then affect the growth manner of precipitates, since it will simultaneously increase the interfacial energy between the precipitates and aluminum matrix (σ) and the diffusion of solute atoms (D).

### 5.2. Role of Dislocations on the Subsequent Aging Precipitation Kinetics of Al-Zn-Mg-Cu Alloy

Although it has been widely discussed that pre-deformation can promote the subsequent aging kinetics of precipitates for aluminum alloys, controversy always exists about whether the dislocation could promote the nucleation of precipitates for aluminum alloys or not. This “controversy” also exists in our calculated results. From Figure 10(d1–d4), it can be found that the precipitate nucleation rate for samples without deformation is obviously higher than those for samples with deformation at whatever aging stage. Especially, at the cooling stage, the precipitate nucleation peak for specimens without deformation occurs earlier than those for specimens with deformation (t1 > t2), as observed in Figure 10(d1). It seems to indicate that the existence of dislocations will inhibit the precipitates’ nucleation of the Al-Zn-Mg-Cu alloy. However, for specimens with deformations, the precipitate nucleation rate increases with the increase in strain at early aging stage, as shown in Figure 10(d1–d3). It seems to indicate that the existence of dislocation will promote the precipitates’ nucleation of the Al-Zn-Mg-Cu alloy. The calculated results seem paradoxical. Of course, it has to be supplemented that it is normal that the precipitate nucleation rate for deformed samples decreases with the increase in the strain at a later aging stage, as shown in Figure 10(d4), since both the growth rate of the precipitates and the consumption rate of the solution in the matrix are faster when the strain is greater. Therefore, the question of how to explain the paradoxical results shown in Figure 10(d1–d3) arises.

**Figure 11:**
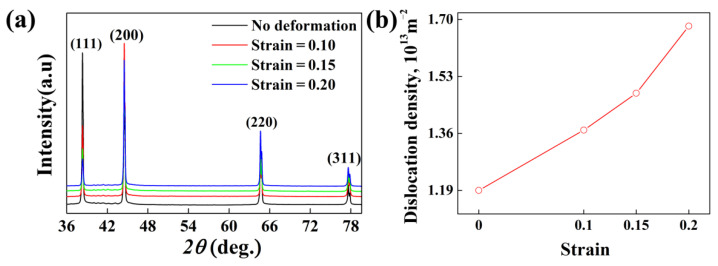
(**a**) XRD patterns; (**b**) the dislocation density of alloy samples with different strains after aging at 120 °C for 28 h.

Here, in order to explore this phenomenon, XRD tests have been carried out to detect the dislocation density in the samples with different strains after water cooling; the results are shown in Figure 11. It shows that the dislocation density increases along with the strain while the maximum density of the dislocations is just about 1.63 × 10^13^ m^−2^, which is one to two orders of magnitude less than that generated by cold deformation (≈10^14^~10^15^ m^−2^) [13,46,47]. According to these calculated results, an interesting hypothesis has been put forward to explain the self-contradictory results shown in Figure 10(d1–d3). The details can be discussed as follows: it is reasonable to put forward that the existence of dislocations can not only promote the nucleation but also the solute atoms diffusion of precipitates; however, it’s necessary to make a supplement for the above conclusions, that is, the actual effects of dislocations on the diffusion of solute atoms and the precipitate nucleation are closely related with the dislocation density ($N_{d}$), which is similar to the influence mechanism of heterogeneous cores on the heterogeneous nucleation of liquid alloys [48,49].

**Figure 12:**
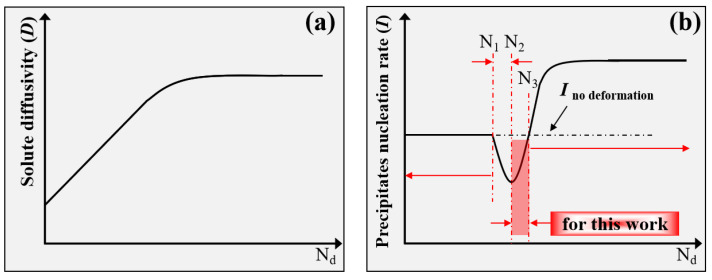
(**a**) The effect of dislocation density ($N_{d}$) on solute atoms diffusion of precipitate; (**b**) the effect of dislocation density ($N_{d}$) on nucleation of precipitate.

In detail, the actual promoting effect of dislocations on the diffusion of solute atoms shows a tendency of increasing and then keeping constant with the increase in $N_{d}$ according to Figure 2b, as shown in Figure 12a. However, for the actual promoting effect of dislocations on precipitate nucleation, when the $N_{d}$ is less than $N_{1}$, it will have no effect on the nucleation of precipitates; when $N_{d}$ is located between $N_{1}$ and $N_{2}$, it will inhibit the nucleation of precipitates and the nucleation rate of precipitates decreases with the increase in $N_{d}$; while when $N_{d}$ is located between $N_{2}$ and $N_{3}$, it will also inhibit the nucleation of precipitates and the nucleation rate of precipitates increases with the increase in $N_{d}$, which is consistent with the results shown in Figure 10(d1–d3). Finally, when $N_{d}$ is more than $N_{3}$, it will promote the nucleation of precipitates, and the nucleation rate of precipitates increases and then keep approximately constant along with $N_{d}$ [7,13], as shown in Figure 12b.

The theoretical values of $N_{1}$, $N_{2}$ and $N_{3}$ are determined by the nucleation ability of precipitates during aging for different heat-treatable aluminum alloys. The actual promoting effect of dislocations on precipitate nucleation is then determined by the actual dislocation density, which is obviously affected by the initial state of raw material and the deformation conditions, such as deformation amount, deformation temperature, deformation method and so on.

If one takes the experimental results in this work for example, the maximum dislocation density is about 1.63 × 10^13^ m^−2^, generated during thermal deformation and then the volume density of the dislocations can be approximately estimated as 2.1 × 10^19.5^ m^−3^, which is just smaller than the nucleation rate (≈2.2 × 10^21^ m^−3^·s^−1^) of precipitates for specimens without deformation. Thus, the existence of dislocation produced by thermal deformation might inhibit the nucleation of precipitates, as shown in Figure 10. Based on the calculation results in Figure 10, it can be deduced that, if the dislocation density can reach 1.7 × 10^14^ m^−2^ or approximately estimated as 2.2 × 10^21^ m^−3^, then it may effectively promote the nucleation of precipitates for the Al-Zn-Mg-Cu alloy. For example, larger cold deformation might promote the nucleation of precipitates of aluminum alloys [7,8,13,16,50], especially for 2XXX aluminum alloys, which has been demonstrated by many other researchers [7,14,16,51]. We can also say that the nucleation ability of the precipitate itself for 7XXX aluminum alloys or Al-Zn-Mg-Cu alloys is too strong to be further catalyzed by dislocations generated by small cold deformation or general thermal deformation [21]. It even probably inhibits the actual nucleation of precipitates for the Al-Zn-Mg-Cu alloy when the dislocation density is not enough [15].

Of course, except for the dislocation density, the dislocation distribution morphology may also affect the precipitate nucleation [52], although this point is not the focus for the above discussion. The above discussion and analysis are based on the assumption that the deformation is approximately homogeneous for the tested samples, that is to say, the dislocation distribution morphology is basically homogeneous. So, the actual situation for the effect of dislocations on precipitate nucleation of aluminum alloys is quite complicated considering that the deformation might be uniform, causing uneven distribution of dislocations and then uneven distribution of precipitates [16,18,52].

### 5.3. Influence of Pre-Deformation on the Microhardness of Al-Zn-Mg-Cu Alloy at Peak Aging

In generally, the contribution of precipitate strengthening to the yield stress of aluminum alloys ($\sigma_{p}$) can be obtained by the following equation [53]:

(11) $$\sigma_{p}=\left\{ \begin{array}{l} \sigma_{p,0}\sqrt{\frac{f_{v}}{f_{v0}}},r\leq r_{0}\\ \sigma_{p,0}\frac{r_{0}}{r},r>r_{0} \end{array}\right.$$

where r is the precipitate mean radius at a given aging time and temperature, $r_{0}$ is the precipitate average radius at peak aging, $f_{v0}$ and $\sigma_{p,0}$ is the precipitates’ volume fraction and the precipitation hardening component at peak aging, respectively. $r_{0}$, $f_{v0}$ and $\sigma_{p,0}$ take values as constants, which mainly depend on the aging temperature.

**Figure 13:**
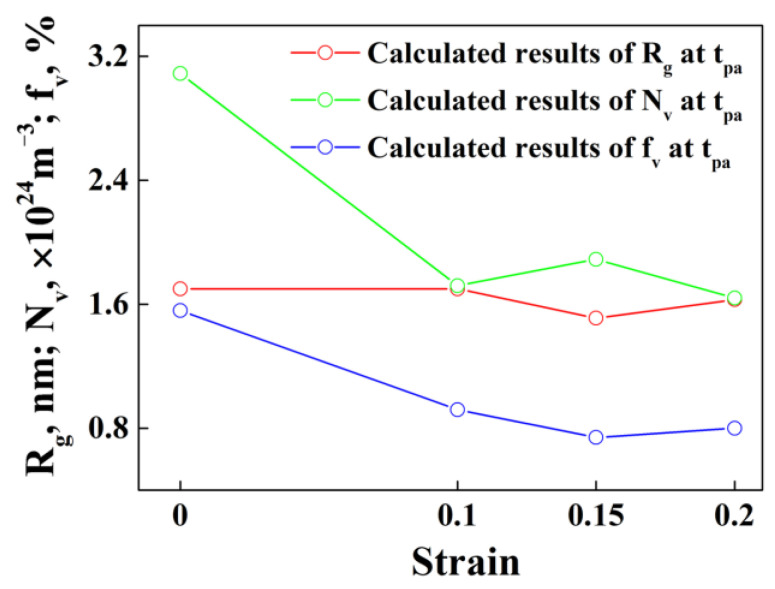
Average radius, number density and volume fraction of precipitates at peak aging ($t_{pa}$) for alloy samples with different strains.

From Equation (11), we can observe that the contribution of precipitate strengthening to the yield stress of aluminum alloys is mainly controlled by the volume fraction of precipitates before peak aging and then the average radius of precipitates after peak aging, rather than the precipitate number density. In order to analyze the influence of aging precipitation under the effect of thermal deformation with different strains on the microhardness of the Al-Zn-Mg-Cu alloy at peak aging, the average radius, number density and volume fraction of precipitates at peak aging for samples with different strains has been collected according to Figure 9, as shown in Figure 13. It shows that, at peak aging, the calculated average radius (≈1.6 nm) of precipitates for specimens with different strains remains constant, while the volume fraction of the precipitates decreases and then increases with the strain. It means that the contribution of precipitate strengthening to the microhardness of the Al-Zn-Mg-Cu alloy will also decrease and then increase along with the strain, since the contribution of precipitate hardening accounts about 80% of the yield stress [54], which is consistent with the experimental results, as shown in Figure 2. This also indirectly proves the reliability of the calculated results.

From Figure 13, it can be inferred that, even though dislocations produced through deformation can affect the nucleation behavior, and then affect the growth manner and growth rate of precipitates, it may not greatly improve or decrease the mechanical properties of the Al-Zn-Mg-Cu alloy, since the deformation condition might be not so perfect and it may not obviously change the stable distribution of precipitates when it reaches peak aging because of the competition for solute atoms between nucleation and growth of the precipitates.

## 6. Conclusions

In order to investigate the influence of thermal pre-deformation on the subsequent aging precipitation for the Al-Zn-Mg-Cu alloy, microhardness tests and microstructure characterizations via SAXS, TEM and XRD have been carried out after thermal simulation experiments and artificial aging treatment. A model has been developed to describe the kinetic behavior of the precipitates and fitting calculations have been carried out based on microstructure characterizations. The main conclusions can be summarized as follows:

(1) As the strain changes from 0 to 0.10, 0.15, 0.20 and 0.25, the peak aging microhardness of the alloy samples changes from 177.7 HV to 166.2 HV, 166.2 HV, 178.2 HV, 181.2 HV at aging times of 17 h, 11 h, 8 h, 7 h and 7 h, respectively, which indicate that thermal pre-deformation can shorten the peak aging time with the peak aging strength being guaranteed.
(2) The calculation results demonstrate that, at peak aging, the precipitate average size almost remains unchanged, while the precipitate volume fraction shows a tendency to decrease and then increase with the strain changing from 0 to 0.10, 0.15 and 0.20, which is consistent with the change in microhardness of the Al-Zn-Mg-Cu alloy samples.
(3) SAXS and TEM results, as well as the calculated results, demonstrate that thermal pre-deformation slightly inhibits nucleation and obviously promotes the growth of precipitates for the Al-Zn-Mg-Cu alloy during subsequent aging, thus further affecting the growth manner of precipitates.
(4) The XRD results and the calculated results for aging precipitate kinetics indicate that dislocations can promote the nucleation and growth of precipitates, while the actual effect of dislocations on the precipitate nucleation and growth might be closely related with the dislocation density.

## Figures and Tables

**Table 1:** Chemical compositions of AA7075-T6 aluminum alloy.

Material	Zn	Mg	Cu	Fe	Si	Mn	Cr	Ti	Al
AA7075	5.39	2.62	1.5	0.50	0.40	0.30	0.23	0.20	Rem

**Table 2:** Other deformation and aging parameters.

Strain Rate (s^−1^)	Strain	Aging Time (h)
0.1	0, 0.10, 0.15, 0.20, 0.25	2, 6, 8, 9, 11, 14, 16, 17, 18, 21, 24, 26, 28

**Table 3:** Main parameters used in the model.

Parameter	Significance	Value	Origin
σ	interfacial energy between the precipitate and the Al-rich matrix	0.30 Jm^−2^	[23,26]
$A_{0}$	constant for the dissolution enthalpy of the precipitate	3.85	[40,41]
$D_{0}$	pre-exponential factor for the diffusion coefficient of solute Zn	6.2 × 10^−4^ m^2^·s^−1^	[37] + this work
$Q_{d}$	diffusion activation energy of Zn	129.3 kJ/mol	[37]
μ	ratio between diameter and thickness of the disc-shaped precipitated phase	3.2	[26,40,42] + this work
R	gas constant	8.314 Jmol^−1^·K^−1^	/
$k_{B}$	Boltzmann’s constant	1.38 × 10^−23^ J/K	/
a	average lattice parameter of the nucleus and the matrix	0.451 nm	/
α	constant	1.05	[23]

## Data Availability

The data used to support the findings of this study are available from the corresponding author upon request.
